# Towards a New Concept of Regenerative Endodontics Based on Mesenchymal Stem Cell-Derived Secretomes Products

**DOI:** 10.3390/bioengineering10010004

**Published:** 2022-12-20

**Authors:** Luis A. Costa, Noemi Eiro, Andrea Vaca, Francisco J. Vizoso

**Affiliations:** Research Unit, Fundación Hospital de Jove, 161, 33920 Gijón, Spain

**Keywords:** dentinogenesis, conditioned medium, MSC, DPSC, dental pulp, cell-free therapy in odontology, regenerative endodontics

## Abstract

The teeth, made up of hard and soft tissues, represent complex functioning structures of the oral cavity, which are frequently affected by processes that cause structural damage that can lead to their loss. Currently, replacement therapy such as endodontics or implants, restore structural defects but do not perform any biological function, such as restoring blood and nerve supplies. In the search for alternatives to regenerate the dental pulp, two alternative regenerative endodontic procedures (REP) have been proposed: (I) cell-free REP (based in revascularization and homing induction to remaining dental pulp stem cells (DPSC) and even stem cells from apical papilla (SCAP) and (II) cell-based REP (with exogenous cell transplantation). Regarding the last topic, we show several limitations with these procedures and therefore, we propose a novel regenerative approach in order to revitalize the pulp and thus restore homeostatic functions to the dentin-pulp complex. Due to their multifactorial biological effects, the use of mesenchymal stem cells (MSC)-derived secretome from non-dental sources could be considered as inducers of DPSC and SCAP to completely regenerate the dental pulp. In partial pulp damage, appropriate stimulate DPSC by MSC-derived secretome could contribute to formation and also to restore the vasculature and nerves of the dental pulp.

## 1. Introduction

The oral cavity is a gateway to the human body and the general health of an individual, so the teeth are key structural and functional elements of this cavity. They are essential for the initial processing of food, for effective speech, and are also a very important factor for correct facial aesthetics, as well as for interpersonal and psychosocial health and well-being. Due to their morphology and tissues that compose them, teeth represent functioning structures of the oral cavity with unique characteristics in the human body. In them, a series of complex and dynamic interactions take place between their different cell types: odontoblasts, immune cells, fibroblasts, endothelial cells, nerves, and pulpal stem cells. But the tooth can be affected by a series of pathologies, among which are mainly caries, periodontal disease, trauma or leakage due to therapeutic procedures. These processes, in turn, can lead to a chain of events such as loss of enamel and dentin, infection, pulpitis, pulp necrosis, and ultimately tooth loss.

The loss of teeth can lead to physical and mental trauma, considerably reducing the quality of life of the subject. More than 50% of the population aged 70 and over are partially or totally toothless [1]. With an aging population, it is estimated that by 2030 more than 50% of people will be 65 years or older. Therefore, the problem of tooth loss is expected to increase in the population [2]. Already in 2006, and without taking into account the recent substantial increase in the age of the population, the journal Dental Economics reported that >30 million Americans were missing all of their teeth in one or both jaws [3] which gives an idea of the socio-sanitary and economic dimensions of the problem.

The alternatives usually employed that arise in this health and social situation are replacement therapies, such as endodontics for damaged pulp or implants for missing teeth. Of fact, for example, each year, around 22.3 million endodontic procedures are performed in the USA alone [4]. Considering the limitations of these procedures, in this review we analyse the possibility of regenerative endodontics based on mesenchymal stem cell-derived secretomes products. For this propose, we revised all published report in Pubmed during the last 25 years on the followed key words: regenerative endodontic, dental pulp tissue engineering, mesenchymal stem cells from the oral cavity and cell-free regenerative endodontic. In addition, we proposed a new concept of regenerative endodontic therapy based on products derived from the secretome from mesenchymal stem cells.

## 2. Dental Pulp Tissue Engineering

The therapy methods currently used to repair pulpal damage and tooth loss are based on replacement materials for restoring structural defects.

In necrotic teeth with immature roots, the possibility of restoring their full functionality is an attractive goal, since the regeneration of lost tissues improves the quality of life of patients [5]. The continuous advances in scientific knowledge have as a practical purpose the development of regenerative therapies of the dental pulp by tissue engineering.

Tissue engineering is an interdisciplinary science that combines engineering and biology principles to generate functional substitutes for damaged tissue [6]. In this field of science, three essential components interact: progenitor cells, inducing biomolecules and a scaffold [7,8]. In the search for a new fully functional dental pulp, various approaches have been developed such as the use of biomaterials, living cells, growth factors and, more recently, mesenchymal stem cell (MSC)-derived products. These new technologies have the potential to change the therapeutic scenario in regenerative dentistry. Therefore, new questions arise about what is the most effective way to apply them to obtain the desired results. For this reason, many studies have focused on the development of personalized scaffolds for pulp tissue engineering, optimizing characteristics and performance [9,10].

A scaffold is basically a natural, synthetic or mixed product that serves as a transport, containment and delivery of bioactive elements to obtain a desired effect [11]. Therefore, it follows that every scaffold must mimic the fundamental characteristics of the native extracellular matrix (ECM) in order to promote and regulate regenerative cellular events [10,12]. Currently, natural biomaterials such as collagen or fibrin are widely used in periodontal guided tissue regeneration, but they are difficult to apply in the reduced endodontic space. For this reason, polymer-based synthetic hydrogels have aroused great interest as custom-made scaffolds suitable for insertion into root canals. As a basic condition within the root canal, a hydrogel must be biocompatible, degraded by the host tissues, and its physical properties and mechanical resistance must match those of the tissue to be regenerated [10,13]. The framework or polymeric network of the hydrogels is customizable and can be obtained, for example, by gas foaming techniques, as well as leaching with salts, to obtain macroporous scaffolds. Whereas, nanofibrous scaffolds have been processed by electrospinning, self-assembly, and phase separation [9,14].

Cell cultures on self-assembling peptide hydrogels have been shown to result in high cell viability [15] and even significantly better results are reported with natural scaffolds [9]. Recent studies suggest that bovine decellularized pulp extracellular matrix could be a promising scaffold in dental pulp regeneration [12].

In addition, given the difficulty of obtaining new vascularization throughout the root canal through the small foramen, there are potential supportive scaffolding strategies for the growth of new dental pulp in the root canals. Thus, for example, there is the possibility of transplanting into the human tooth without pulp a cell-based vascularized construct, a prospective biomaterial as an implant for the developing dental pulp, thus facilitating blood supply and improving pulp regeneration [16]. Scaffolds can also be preloaded with antimicrobials to optimize disinfection as a prerequisite for dental pulp regeneration. An in vivo study using poly (vinyl alcohol)-chitosan nanofiber scaffold with ciprofloxacin and synthetic cationic peptide IDR-1002, had anti-biofilm activity against *Enterococcus faecalis*, *Staphylococcus aureus*, and a multispecies oral biofilm [17].

To date, research on pulp tissue regeneration through tissue engineering has not yet fully restored biological functions, such as promoting blood vessels and nerve supply. Thus, the ideal would be the implementation of regenerative endodontics procedures (REP), fundamentally based on the fact that the oral cavity is a large source of mesenchymal stem cells (MSC).

## 3. MSC of the Oral Cavity

**Figure 1:**
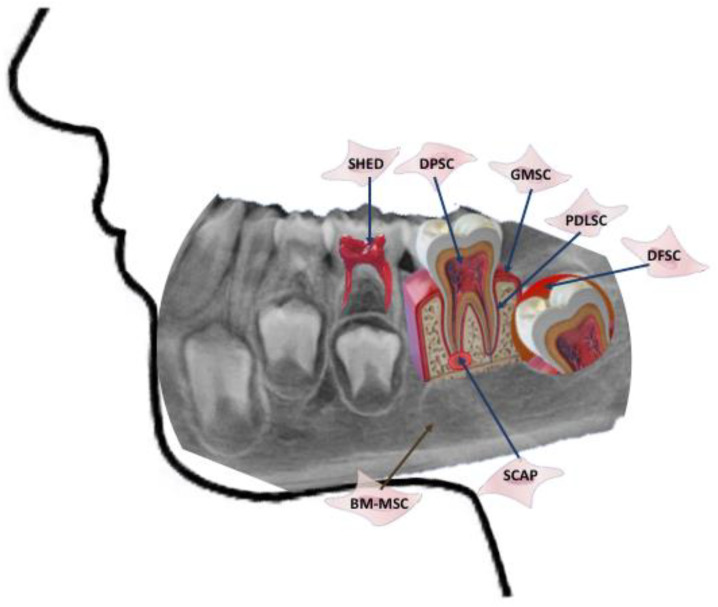
Sources of MSC from oral cavity. SHED: stem cells from human exfoliated deciduous teeth; DPSC: dental pulp stem cells; GMSC: gingiva-derived mesenchymal stem cells; PDLSC: periodontal ligament stem cells; DFSC: dental follicle stem cells; BM-MSC: bone marrow mesenchymal stem cells; SCAP: stem cells from apical papilla.

The first group of stem cells of buccal origin was isolated from human dental pulp and known by the name of dental pulp stem cells (DPSC) [18,19]. Subsequently, other MSC have been isolated from oral tissues, receiving names according to the source of origin: pulp tissue of exfoliated primary teeth (SHED) [20]; periodontal ligament (PDLSC) [21]; apical papilla of developing teeth (SCAP) [22]; dental follicle (DFSC) [23]; gingiva (GFSC) [24] and buccal fat pad (BFPSC) [25]. The MSC of the tissues that make up the oral cavity have the main function of maintaining its integrity and homeostasis, as well as its repair in case of injury [26] (Figure 1).

### Postnatal Dental Pulp Stem Cells

Teeth are considered a possible source of MSC, due to their relatively easy access and even with reports of isolation of pulps affected by caries [27], pulpitis [28,29,30], and third molars [31,32]. In addition, due to the unique ability to regenerate dental tissue, DPSC have been of great interest in various medical applications due to their characteristics. There are reports indicating that DPSC maintain their genomic stability even after multiple passages in vitro [33]. This cell express markers typical of MSC with proliferative and differentiation capabilities across multiple lineages, even when they come from inflamed pulp. Although it was observed that in such circumstances they seem to be dysfunctional, since they present a decrease in immunomodulatory capacity and in their osteogenic or dentinogenic potential [34,35].

DPSC represent a very small group of cells located generally in the central zone of the pulp, whose number decreases as the individual ages [36,37]. Although there is no consensus in the literature about the methods of isolation and expansion of DPSC. The most widely used methods for obtaining DPSC are (i) from pulp of exfoliated primary teeth or extracted permanent teeth in a sterile environment, or (ii) performing a pulpotomy preserving the tooth. The most widely used obtaining method for DPSC is from pulp tissue of extracted third molars [31,38]. Pulpal MSC present a fibroblast-like morphology under the microscope and they express the typical surface molecules that characterize MSC [19,39,40].

In cultures, DPSC showed a high level of colony formation and proliferative capacity, including differentiating into various cell types. Particularly, DPSC have the potential to osteogenic differentiation [41], and to differentiate into dentin-forming odontoblasts [42] as well as neurogenic capability, perhaps due to their neural crest origin [43]. When DPSC were co-cultured with trigeminal neurons, high levels of neurotrophin expression demonstrated the neurogenic potential of these cells [44,45]. Interestingly, it has been showed an enhanced effect of ascorbic acid on differentiation, secretome and stemness of DPSC [46]. On the other hand, similarly to other MSC, DPSC exhibit immunomodulatory activity [19,47] by releasing cytokines and growth factors with paracrine action into their environment. They carry out paracrine action both in immunoregulatory functions and in the regeneration of pulp damaged tissues, which makes them a therapeutic strategy to consider in the context of cell therapies [48,49,50,51,52].

## 4. Types of Regenerative Endodontics Procedures (REP)

On the basis of the presence of DPSC in dental pulp, there are two alternatives to REP: cell-based REP and cell-free REP (without exogenous cell transplantation).

### 4.1. Cell-Based REP

Cell-based REP consists of the implantation of autologous or exogenous DPSC inside the root canal, so that they proliferate and differentiate in the different cellular phenotypes of the dental pulp [53]. In dentistry, cell-based REP using exogenous DPSC or SHED have been considered a good alternative, which combined with a custom scaffold, the result is the regeneration of pulp-like tissues [32]. Preclinical studies allow translation from the laboratory to the living being, for a better understanding of the interaction of the host with the scaffolds and their biological content. In vivo studies can be based on ectopic, semi-orthotopic and orthotopic approaches, which have their advantages and disadvantages. Orthotopic regenerative studies with procedures similar to those used in humans but using animal teeth are the most relevant, since they allow evaluating the safety and efficacy of the new approach, and provide information on the changes or adjustments necessary for future clinical protocols. [54,55]

A number of case reports suggest that DPSC can be used for the treatment of intra-bone defects. In these patients with bone defects caused by periodontal disease, collagen sponges and autologous/allogeneic DPSC have been applied through minimally invasive surgeries. The results showed decreased probing depth, new fiber reinsertion, and new bone formation with few adverse effects, indicating that DPSC have regenerative potential for periodontal tissue [56,57]. DPSC have the potential for neurogenic, angiogenic, and odontoblastic differentiation and are therefore considered the main contributor to complete pulp regeneration. In addition, some studies have shown that transplantation of cultured stem cells is effective in the regeneration of the dentin-pulp complex. Studies in vivo, demonstrate that it has been possible to form a tissue similar to dentin by means of DPSC and hydroxyapatite/tricalcium phosphate (HA/TCP) scaffolding, presenting a layer of cells similar to odontoblasts that expressed dentin sialophosphoprotein (DSPP), a specific marker related to with differentiation into odontoblasts [58,59].

The first report on regeneration of the dentin-pulp complex in large animals was made by Iohara et al. [29] who got it by implanting canine pulp-derived stem/progenitor cells into pulpotomized teeth in dogs. The same group reports complete pulpal regeneration after pulpectomy in dogs by autologous transplantation of a combination of CD105 (+) pulpal stem cells with stromal cell-derived factor-1 [60].

In in vitro and in vivo studies in animal models, showed that SHED combined with a self-assembly peptide scaffold like Puramatrix™ (BD Biosciences, Bedford, MA, USA) or Collagen type I (human recombinant BD™ Fibrogen Collagen Type I; BD Biosciences) presented similar cellularity and vascularization when compared with control human dental pulps [61].

A pilot clinical study in five patients with irreversible post-traumatic pulpitis in permanent incisors, G-CSF-pretreated DPSC were implanted into the empty root canal, observing in 3 patients a vascularized and neural reconstruction of the pulp tissue with new dentin formation at 24 months of follow up [30]. In a randomized clinical trial in teeth with necrotic pulps, they obtained complete regeneration of all tissues of the dental pulp, with connective tissue, blood vessels, odontoblasts arranged in layer and neuronal markers after transplantation of DPSC in situ [62]. Nevertheless, there are relatively few studies reporting the safety use of MSC. Recently was informed the regenerative potential of the pulp tissue and the safety of DPSC transplantation in pulpectomized teeth in dogs [63] and clinical trials [64], without showing toxicity or subsequent adverse events. Other studies indicate the efficacy and safety of MSC from other origins, such as allogenic umbilical cord MSC which have been proved in a preclinical trials [53,65]. However, it is likely that DPSC were not good candidates for periodontal regeneration due to their limited ability to form cementum [66,67].

On the other hand, we also have to mention the possibility of obtaining cells and odontoblastic potential through other origins. Thus, it is of interest the finding that BMP-4 and FGF-8, induces human embryonic stem cell differentiation into odontoblastic lineage [68]. In addition, the induced pluripotent stem cells (iPSC) have capability for indefinite self-renewal and large-scale expansion. In vitro and in vivo studies using iPSC from DPSC with proper stimulation can differentiate into fully functional odontoblasts [69]. In fact, it has been shown that iPSC can be used for test the bioactivity of materials and other regenerative therapies including the treatment of dentin and/or dental pulp damage [69,70].

#### Limitations of the Cell-Based REP

There are several drawbacks in relation to stem cell-based REP, which make it difficult to transfer this technology to daily clinical practice.

The main drawback comes from the isolation and expansion of the cells, since healthy teeth must be provided by donors or, it is necessary to store them in a cell bank [71]. Furthermore, obtaining enough MSC in 2D or 3D cultures for regenerative treatments is time-consuming, expensive, and usually requires a specialized laboratory [54,72]. To this fact it is necessary to highlight recent reports that implanted MSC do not survive long [73,74,75]. Evidence demonstrates that MSC transplantation results in a low rate of engraftment at the target site. Inflamed tissues are a hostile environment that reduces the survival of MSC to a half-life of 24 h [76]. As an example, injection of SHED into a bone defect due to periodontitis diffused slightly for 3 days post-grafting and then decreased rapidly [77]. It follows that the injection of a high number of MSC is not related to the beneficial results observed [78].

### 4.2. Cell-Free REP

There are alternatives for REP by using cell-free strategies, such as based on either blood or MSC derived products.

#### 4.2.1. Cell-Free REP Based on Blood Derived Products, Bioactive Molecules or Bioingenniering Materials

Within cell-free REP strategies there are two therapeutic procedures such as pulpal revascularization and active induction of local cells. The first evidence of attempts to regenerate pulpal tissue dates back to the 1960s by Nygaard-Ostby [79], whose research in animal models and in humans describes an alternative treatment for the regeneration of dental pulp in permanent teeth. This consists in a certain way similar to what we currently known as revascularization, through a blood clot inside the root canal. Subsequently, alternative scaffolds have been proposed to replace the blood clot, such as autologous platelet-rich plasma (PRP) and platelet-rich fibrin (PRF) and take advantage of its regenerative potential [80] to resolve apical periodontitis and complete root development [81,82]. A REP standard protocol was proposed by the American Association of Endodontics (AAE) in 2016 [83]. This strategy includes a multi-step procedure: control of the infection of the affected tooth, stimulation of bleeding of the periapical tissues by gentle irritation with a pre-curved K-file passing 1–2 mm through the apex, and to perform a blood clot in the root canal, which serves as a suitable scaffold for the growth of new tissue. Subsequently, a biomaterial is placed in contact with the blood clot at the cervical level of the root, usually with mineral trioxide aggregate (MTA), followed by the permanent coronal seal to avoid bacterial reinfection [84,85].

Within the cell-free approach, it has been proposed to regenerate pulp-like tissue by means of molecules that induce the recruitment of the patient’s endogenous MSC into the root canal [86,87]. It is believed that this strategy without transplantation of exogenous cells is capable of achieving regeneration of the dentin-pulp complex more effectively than simple revascularization. For this purpose, in preclinical studies several molecules have been used as homing factors, including VEGF, basic fibroblast growth factor (FGF), bone morphogenetic protein-7 (BMP-7), platelet-derived growth factor (PGF), and nerve growth factor (NGF) showing promising results [88]. As a result of these investigations, it can be inferred that cell-free REP show a therapeutic effect on necrotic immature teeth. It has been shown that MSC expressing CD105, CD73 and STRO1 migrate in large numbers into the empty root canal by periapical bleeding [80]. In these studies of necrotic immature permanent teeth treated with the revascularization technique, the MSC found within the canal were expected to come from the adjacent apical papilla, but MSC from other surrounding tissues are probably present. In fact, the newly formed tissues were cementum-like and bone-like, which were confirmed by immunohistochemical staining. Therefore, it is presumed that MSC from the all periapical tissues also entered into the root canal and were co-responsible for the formation of these new mineralized tissues during root maturation [89,90].

There are also several materials that can be used to induce odontoblastic differentiation. Of fact, for now, the gold standard material for vital pulp capping or apexification is MTA, first introduced to the dental market in 1998 [91]. It is also of note that graphene induced osteogenic differentiation of DPSC without the use of chemical inducers for osteogenesis [92]. In addition, in order to promote bone regeneration, growth factors with osteoinductive activity have been used, such as bone morphogenic protein (BMP)-2, embedded in various scaffolds or osteoconductive materials [93,94]. However, there are several issues with the use of some of these factors for tissue regeneration. Thus, for example, recent studies on bone regeneration through the clinical application of BMP-2, have described unexpected side effects [95], such as the induction of a severe inflammatory response, probably due to a higher dose than necessary [96,97,98].

In some case reports it is suggested that cell-free REP based could eliminate apical periodontitis and even revitalize immature necrotic teeth, but their results still inconsistent. When the dental pulp, apical papilla, or follicle are severely damaged, there are no MSC to differentiate into odontoblasts and promote dentin formation, resulting in the interruption of dental root development. True regeneration of the dentin-pulp complex is rarely seen or detected in the root canal [99]. Hence, there is great interest in developing new strategies to achieve pulp regeneration and return its organized structure to the dentin-pulp complex with original homeostatic functions.

#### 4.2.2. A New Concept of Regenerative Endodontics Based on Secretome-Derived Products from Mesenchymal Stem Cells

Accumulated knowledge reveals that soluble factors with paracrine action secreted by MSC are responsible for the observed beneficial effects. Biological products are present in the secretome of the MSC, which contain a large number of bioactive proteins, such as growth factors and cytokines, as well as extracellular vesicles (EV). EV represent an important component of the MSC secretome that has aroused great interest as a true option for therapeutic applications. EV can be categorized as (i) exosomes (30–120 nm in diameter), that come from intracellular multivesicular bodies (MVB) and are released by the fusion of MVB with the plasma membrane; (ii) microparticles (150–1000 nm in diameter), that originate from blebs that emerge from the plasma membrane and are released into the external environment by proteolytic cleavage of the cytoskeleton; and (iii) apoptotic bodies (500–2000 nm in diameter), originating during the process of programmed cell death. Among these microparticles, exosomes aroused great general interest for their role in cell biology and their potential therapeutic and diagnostic applications. Exosomes are secreted by cells to facilitate intercellular communication. This is carried out through their components, such as lipids, several types of RNA, cytokines, chemokines, integrins (CD81, CD63 and CD9), as well as signal transduction factors, transport proteins (annexins and Rab GTPases), cytoskeleton proteins and metabolic enzymes. After the discovery of their role in the transfer of mRNA, miRNA and active factors, exosomes represent a powerful intercellular communication pathway in organ homeostasis and disease. These mechanisms can be achieved by binding to target cell surface receptors that activate signalling cascades, internalization of cell surface-bound exosomes, and direct fusion with the target cell to release its cargo to the cytoplasmic membrane and cytosol [100].

**Figure 2:**
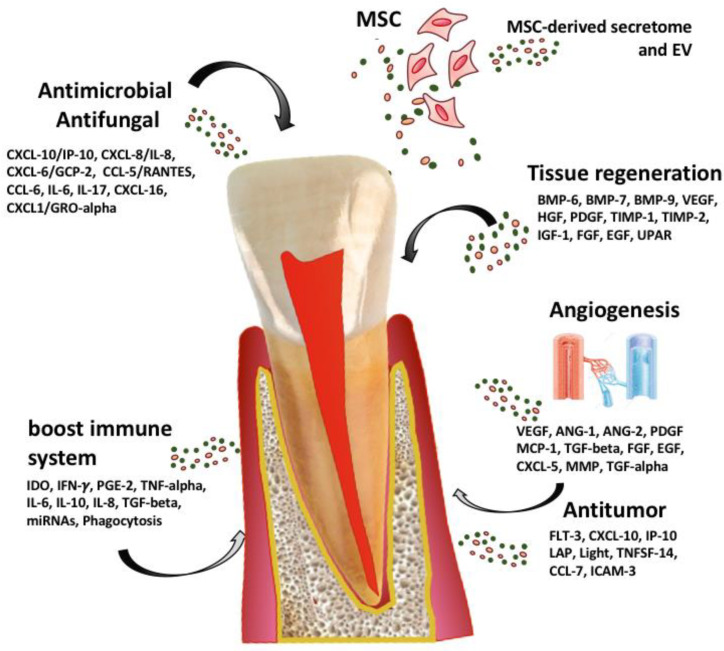
Potential functions and applications of MSC-derived secretome and extracellular vesicles (EV).

MSC-derived secretomes, obtained from culture conditions of MSC and named conditioned medium (CM), can be considered as promising candidates for direct medical biotechnology, due to their anti-inflammatory, angiogenic, anti-oxidative stress, anti-apoptotic and regenerative activity [98,100,101] including for REP (Figure 2).

This cell-free strategy would avoid the drawbacks derived from the graft of proliferating stem cells, among which the following stand out: (i) immunological incompatibility, tumorigenicity, formation of emboli, transmission of infections and potentially entry into senescence or apoptosis; (ii) It is possible to better control the biosafety, dose and potency of a secretome, similar to conventional therapeutic agents; (iii) the lyophilized secretome can be stored without the addition of potentially toxic preservative substances; and (iv) using secretome-derived products, such as conditioned medium or exosomes, is more cost-effective and suitable for clinical use, as it would be “ready to use”. This is related to the fact that a secretome could be available for treatment when needed, being a product prepared in advance [72].

Previous studies using MSC conditioned medium (MSC-CM) have been shown to be safe with less inflammatory signs and appear to have great osteogenic potential for alveolar bone regeneration [102] and for periodontal tissue regeneration [103]. Several studies also revealed that MSC-CM contains diverse cytokines, such as IGF-1, VEGF, TGF-β1, and HGF, that enhances early bone and periodontal tissue regeneration [104,105,106,107]. The growth factor IGF-1 is known to induce osteoblast proliferation and migration [108,109] and stimulate periodontal ligament (PDL) cells through the Pi3 K pathway, enhancing periodontal tissue regeneration [110]. In addition to enhancing endothelial cell survival and differentiation, VEGF is also believed to be involved in the process of osteogenesis [111]. There is another factor that has a direct effect on angiogenesis, such as HGF and TGF-β1 [112]. Among the main functions of TGF- β1 are that of increasing the migration of osteoprogenitor cells, regulating cell differentiation and proliferation, and the production of extracellular matrix [113]. It has been detected that TGF- β1 is expressed during the development of alveolar bone and cement, stimulating the regeneration and repair of the PDL (112) [114].

All this is especially interesting if we take into account that a previous studies showed that the combination of several growth factors would have an additive effect on cell migration and osteogenic differentiation [115,116].

The anti-inflammatory effect can be especially interesting to control inflammation in the context of bone regeneration. In fact, it was shown that only a few inflammatory cells were detected at the site where MSC-CM was placed in an allogeneic animal study on calvarial bone defects [104,105] or periodontal tissue regeneration [106,107].

Interestingly, there are data indicating the regenerative interest of the DPSC secretome. An early report indicates that direct co-culture of DPSC and endothelial cells enhances the in vitro differentiation toward osteogenic-/odontogenic and angiogenic phenotypes, which suggests that those oral stem cells display paracrine mechanisms [117]. More recent in vivo data indicate that DPSC condition medium (DPSC-CM) could efficiently prevent neurodegeneration, neuroinflammation and enhance hippocampal neurogenesis in a mouse model of hippocampal neurodegeneration [118]. In addition, DPSC secretome had higher expression of the cytokines like GCSF, IFNγ, and TGFβ that promote neural differentiation [119].

Apart from the potential beneficial effects on pulp regeneration of the high concentrations of soluble factors in MSC-CM, a large part of these effects may be due to extracellular vesicles (EV) and, especially, exosomes that have been detected in appreciable quantities in those biological media. Experimental studies reveal that exosomes play an emerging role in a wide range of pathologies [47] and even trigger the increased expression of genes required for odontogenic differentiation and regeneration of dental pulp-like tissue [120]. In relation to oral diseases, there is increasing evidence suggesting that exosomes act as a fundamental regulator of homeostasis and the immune response. Therefore, a new therapeutic approach through the application of exosomes derived from oral MSC could represent a great advance in the treatment of different oral pathologies, such as osteonecrosis of the jaw (ONJ), periodontal disease and oral cancer [121,122,123].

In chronic periodontitis, the mechanism of local inflammation and its relationship with systemic conditions is complex, in which exosomes play a key role. In peripheral blood tests of patients with this pathology, an imbalance of T-helper17 (Th17)/T-regulatory (Treg) cells had been detected. PDLSC-derived exosomes from healthy tissue alleviated the inflammatory microenvironment through the Th17/Treg/miR-155-5p/SIRT1 regulatory network. Consequently, in the face of periodontal disease, PDLSC-exosomes make a fundamental contribution to the maintenance of tissue homeostasis [124]. Moreover, during periodontal inflammation PDLSC-exosomes are involved in the regulation of bone remodelling [125]. Anti-inflammatory activity has been reported of PDLSC-derived exosomes during their interaction with macrophages [126].

Regarding SHED-derived exosomes, their effectiveness in stimulating the osteogenic capacity of PDLSCs was observed by activating Wnt/-catenin and BMP signalling pathways [127]. In a mouse model of induced acute lung injury, SHED-exosomes also upregulated the immune response with anti-inflammatory effects [128]. Derived exosomes of the same cellular origin cultured under odontogenic differentiation conditions promoted regeneration of dental pulp-like tissue using both DPSC and bone marrow stem cells in a dental root fragment model, with increased expression of DMP1 and DSPP [120]. GMSC-derived EV have anti-inflammatory potential by downregulating TNF-α through the significant production of interleukin-1 receptor antagonist, during gingival and cutaneous wound healing in mice [129].

Oxidative stress is a condition capable of stimulating inflammation and inducing various oral pathologies, such as periodontal disease. One study indicates that GMSC-derived exosomes reduce entry into cellular senescence of inflamed tissues [130]. In addition, GMSC exosomes promoted wound healing in a diabetic mouse study by stimulating collagen remodelling, angiogenesis, and re-epithelialization [131].

All these data on the biological effects of exosomes may be relevant if we consider the potential advantages of these EV. They are comparatively smaller, their membrane is less complex and contains lower levels of membrane-bound proteins, so they develop less immunogenicity than their parent cells, and their production and storage is also easier than with parent cells.

Recent studies show similar potential beneficial effects of both MSC-CM and EV for pulp regeneration. It has been shows that CM from human DPSC induce osteoblast differentiation in vitro [132], and it was effective in vivo in controlling pulp tissue inflammation and improve dentin bridge formation and its morphological quality [133]. With regard to exosomes derived from human MSC or DPSC, it was proposed their therapeutic effects on the basis of their angiogenic [134,135] and neuroprotective function [120]. The alternative use of CM or Exosomes has advantages and disadvantages. The production of an adequate amount of MSC-derived exosomes in cell cultures is complex, as is their isolation and purufication despite the various technical options for it (centrifugation, ultracentrifugation or size exclusion chromatography) [92]. However, these microvesicles are better defined products with a view to approval by regulatory agencies for their therapeutic use. Even so, future comparative in vivo studies will be necessary in order to select the most powerful and suitable derivative of the MSC secretome for pulp regeneration.

Other possible interest of MSC-CM is in the field of scaffolding. Excitingly, US FDA approved a xenogeneic extracellular matrix (ECM) scaffold for human therapeutic procedures [136], so the biomimetic tooth bud model could be a hopeful treatment for the goal of dental regeneration [137]. Scaffolds preloaded with MSC derivatives be able to stimulate host tissue stem cells to migrate and differentiate thereby contributing to tissue regeneration [138]. Thus, engineering for scaffolds development is constantly evolving in order to provide effective tools for the regeneration of dental tissues. A key factor in the development of preloaded scaffolds is the careful selection of growth factors and cytokines capable of promoting the chemotactic and proliferative activity of host MSC [139,140]. On the other hand, there are many studies focused on the development of optimal material characteristics, such as tailor-made scaffolds for therapeutic applications in dental pulp engineering [9,10,141,142,143]. In cases where part of the dental pulp was intact and vital, adequate induction would result in the mobilization of progenitor cells from it onto a scaffolding material to harbor, proliferate, and differentiate [144]. In a recent review, the method of using scaffolds for odontoblast induction appears to be more convenient than without it [145].

It is widely recognized that, when possible, the best option is the conservation of the tooth with its proprioception versus rehabilitation with dental implants. The exquisite tactile sensitivity of natural teeth, such as biting with the incisors or limiting excessive chewing loads, cannot be replaced by osteointegrated implants [146]. In addition, the tooth has the periodontium that exerts a protective and defensive function, such as the junctional epithelium and the perpendicular insertion of Sharpey’s fibers from the bone to the root cement. These structures are absent in the implant, since on their surface there is only a longitudinal organization of collagen fibers in the area close to the abutment/implant interface, thus being less resistant to microbial aggression [147].

**Figure 3:**
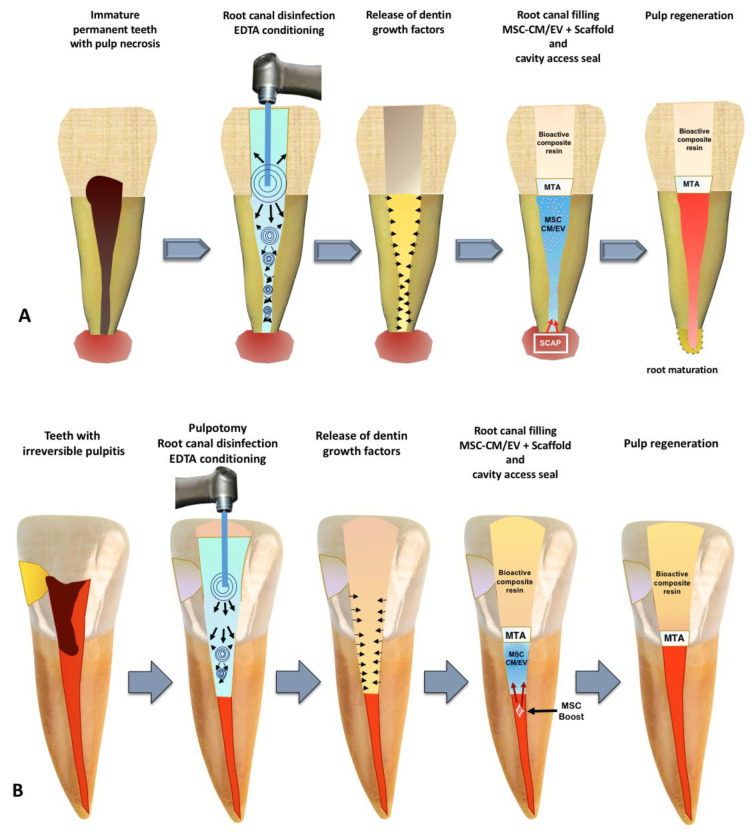
Proposal for a cell-free approach for dental pulp regeneration by using MSC-derived CM/EV. In (**A**) the treatment sequence for immature permanent teeth with pulp necrosis is outlined. In (**B**) the regenerative procedure for cases with partially damaged pulp is summarized. MTA: mineral trioxide aggregate; SCAP: stem cells from apical papilla; EDTA: Ethylenediaminetetraacetic acid.

In this sense, we can consider that, starting from the concept that nothing can overcome nature itself, we propose a new strategy for regeneration of the dental pulp, recovering all anatomical characteristics and functions of the original pulp. This involves new blood supply, innervation and all cell types including dentinogenic ones [148]. This proposed strategy is based on the theory that DPSC are residents close to the perivascular niches in the postnatal dental pulp [27,149] or the SCAP from apical papilla [150] and could be induced by MSC-CM or EV to increase their regenerative potential (Figure 3). In addition, we added a propose on the application of these MSC-derived products into root canals. Of fact, there is the possibility to combine the MSC secretome-derived products with new technologies. Hydrogels are employed due to their biocompatibility, similarity to the extracellular matrix, permeability for oxygen, small molecules and nutrients [151,152], converting them into optimal carriers of MSC secretome to regenerate dental pulp.

On the other hand, furthermore, soluble factors of potential interest in pulpal regeneration have been identified. Thus, it was showed the capacity of DPSC and platelet-rich plasma (PRP) to regenerate dental pulp in canine mature permanent teeth [153]. Recently, hypoxia-inducible factor-1α (HIF-1α) has been found to exert a protective effect on implanted SHED in relation to their survival and angiogenic activity, by regulating reactive oxygen species homeostasis and glycolysis-related gene expression as factors essential in the early post-implantation stages [154,155].

## 5. Conclusions and Future Perspectives

The use of MSC-derived secretome, such as CM or EV, instead of cell therapy can promote the stimulation of proliferation and differentiation of MSC from dental pulp towards odontoblasts, blood vessels and nerves. This concept may represent the basis for a new regenerative endodontics strategy. For this purpose, and due to their multifactorial biological effects, MSC-derived secretomes might be considered from non-dental sources. In addition to more often used sources such as adipose tissue, bone marrow or UC, other secretomes sources are having interest. Thus, for example, both human GMSC [156,157,158,159], or uterine cervical-MSC (hUCESC) [160,161,162] secretomes may be good candidates as regenerative inducers in cell-free REP due their potent regenerative and anti-inflammatory biological effects. Besides, hUCESC are easy to isolate and culture, and proliferative faster than other MSC, such as bone marrow mesenchymal stem cells (BM-MSC) [163] or adipose derived stem cells (AD-MSC) [164]. Nevertheless, further studies should be carried out to explore this hypothesis, as well as to develop methods directed to adequate clinical applications of MSC-derived secretomes, such as their well standardized and mass production and in vitro in bioreactors, as well as the use of functional assays to test the obtained biological products and appropriate experimental models to explore the mechanisms [165].

## Data Availability

Not applicable.
